# Legacy effect of statins: 20-year follow up of the West of Scotland Coronary Prevention Study (WOSCOPS)

**DOI:** 10.21542/gcsp.2016.35

**Published:** 2016-12-30

**Authors:** Mohammed Amin Kashef, Gregory Giugliano

**Affiliations:** 1 Division of Cardiovascular Disease, Baystate Medical Center, Springfield, MA, USA; 2 Tufts University School of Medicine, Department of Medicine, Boston, MA, USA

## Introduction

The West of Scotland Coronary Prevention Study (WOSCOPS) was a randomized, placebo-controlled, primary prevention trial of pravastatin in men aged 45 to 64 (mean age of 55 years) with no history of myocardial infarction at randomization. A total of 6,595 men, with a mean (SD) plasma cholesterol level of 272 (23) mg/dL and mean (SD) low density lipoprotein cholesterol (LDL-C) of 192 (17) mg/dL were randomly assigned to receive pravastatin 40 mg daily or placebo for five years. The primary outcome was a composite of death from coronary heart disease (CHD) and nonfatal myocardial infarction. There was a 31% relative reduction in the primary outcome with pravastatin. There was similar reduction in risk of nonfatal myocardial infarction, death from CHD and death from all cardiovascular causes with no increased risk of death from non-cardiovascular causes nor an increase in incident cancers^1,2^.

Post-trial extended follow-up for 10 years showed continued reduction in coronary events^3^ and follow-up for 15 years showed reduction in healthcare resource utilization and cost, in addition to increase in quality of life^4^. Despite these findings, use of statins in the primary prevention of cardiovascular disease has been hotly debated in the literature^5,6^. Twenty-year follow-up of WOSCOPS was designed to provide information on the long-term outcome, safety and economic benefits of pravastatin therapy for primary prevention of cardiovascular disease^7^.

## Description of the study results

In the original WOSCOPS trial, subjects were enrolled from 1989 to 1991 with the final follow-up visits in 1995. The rate of statin use after trial completion and at 5 year follow-up was relatively low (38.7% and 35.2% statin use in the pravastatin and placebo groups, respectively). Extended follow-up of events for 20 years (until 2011) was performed through record linkage from death and cancer registries and all hospitalizations from national electronic hospital discharge records.

Information on outcomes including incident cancers, CHD, heart failure, stroke, coronary revascularizations and hospital admissions were collected. Outcome differences were attributed to the 5-year treatment allocation in the original trial. The impact on mortality, incident cancers and the cumulative number of hospital admissions over 20 years or until death was assessed. Cumulative event rates were calculated to assess the impact of statin therapy on the total burden of disease and healthcare resource utilization.

**Figure 1.**
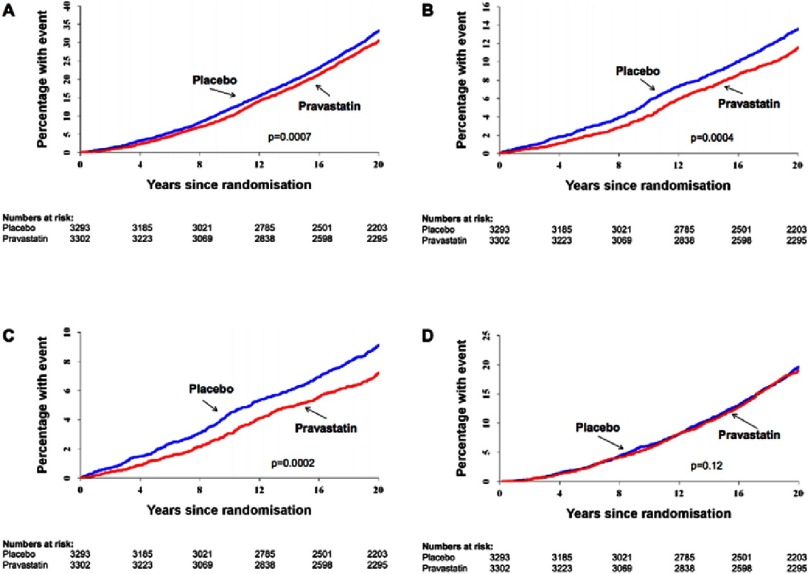
Cumulative mortality from (A) all causes, (B) cardiovascular disease, (C) coronary heart disease, and (D) non-cardiovascular disease. *P* values were determined by Cox proportional hazards model.^7^

All-cause mortality rate was reduced from 38 % in the placebo group to 34.7 % in the pravastatin group (HR = 0.87, 95% CI 0.80–0.94, *P* = 0.0007). Cardiovascular and CHD mortality rates were also reduced (HR = 0.79, 95% CI 0.69–0.90, *P* = 0.0004 and HR = 0.73; 95% CI 0.62–0.86; *P* = 0.0002, respectively) (Fig. 1). Mortality from stroke, non-cardiovascular causes and cancer did not change significantly. Moreover, there was no evidence of increased incident cancer.

**Figure 2.**
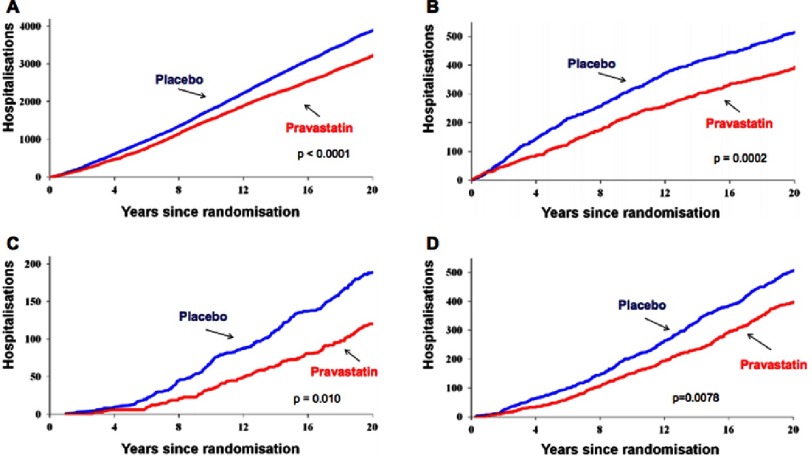
Cumulative numbers of hospital admissions.^7^

A reduced total burden of disease was evident by reductions in cardiovascular admissions and admissions involving coronary revascularization in the pravastatin group, as well as an 18% reduction in admissions due to recurrent coronary events, 24% reduction in myocardial infarction and 35% reduction in heart failure admissions. Continuing divergence of the cumulative event curves was observed, as depicted in Fig. 2. In further analysis, the pravastatin group had an increased risk of day admissions for cataract surgery and lower rate of non-cardiovascular hospitalization due to diabetes complications. No significant impact on stroke admissions or admission for non-cardiovascular causes was observed.

## Discussion and critiques

The latest guidelines from the American College of Cardiology/American Heart Association on blood cholesterol management assign a class I recommendation to primary prevention with statin therapy in adults ≥21 years of age with LDL-C ≥190 mg/dL; diabetic patients aged 40–75 years with LDL-C of 70–189 mg/dL; and non-diabetics who have an estimated 10 year risk of ≥7.5% based on Pooled Cohort Equations. Furthermore, statin therapy for primary prevention should be individualized based on the cardiovascular risk/benefit profile, the potential for adverse effects, drug-drug interactions and patient preferences. The absolute benefit in risk reduction depends on the baseline absolute risk for cardiovascular disease; hence, patients with higher baseline risk will benefit more from treatment^8^.

WOSCOPS was published in 1995 and studied men with a mean age of 55 years and mean LDL-C of 192 ± 17 mg/dL. Participants were reported to be representative of typical middle aged men with hypercholesterolemia and no history of established cardiovascular disease. Five years of pravastatin therapy was compared with placebo. Pravastatin was well tolerated and resulted in no more study withdrawal than placebo^2^. Twenty-year follow-up of WOSCOPS gave a unique opportunity to examine the long-lasting effect of statins in a stable population and suggested that 5 years of treatment with pravastatin provided a legacy effect over a 20-year period^7^. This might point to a lifetime benefit, as age 55 to 75 years covers the entire period of premature cardiovascular morbidity and mortality.

No other major statin trial has come close in terms of length of follow-up as the 20-year follow-up of WOSCOPS. This long term follow-up allowed for a unique assessment of outcomes not typically evaluated in a cohort of patients with hypercholesterolemia and no established CHD. One such example was the remarkable finding of a 35 % risk reduction in heart failure hospitalizations in those patients assigned to pravastatin compared to placebo.

Twenty-year follow-up of WOSCOPS did not show a reduction in stroke mortality or hospitalization. Association between cholesterol level and risk of stroke is hotly debated in the literature. Observational studies have suggested that cholesterol lowering with statins may not be effective in primary prevention of stroke for low risk patients without preexisting CHD^9^, a finding that is in contrast to the results of other trials. The Cholesterol Treatment Trialists’ (CTT) Collaborators conducted a meta-analysis of 27 trials involving 175,000 participants who were at low risk of vascular events. They found a reduction in all major vascular events including any stroke in patients with 5-year risk of an event lower than 10%. The CTT concluded that each 1.0 mmol/L (38.7 mg/dL) reduction in LDL-C will result in 11 fewer major vascular events per 1000 over 5 years^6^. Due to the very low rates of stroke in these primary prevention trials, demonstrating a significant statistical reduction in the first occurrence of stroke has been elusive. However, stroke reductions have been shown is certain primary prevention studies with a variety of statins in selected populations^10–12^.

Side effects of statins have been widely reported, including myopathy^6,13^ and an increased risk of diabetes^6,14,15^. Although an association between statin therapy and increased risk of developing diabetes has been shown, the benefits of statin therapy in diabetic patients to reduce their risk of cardiovascular events is well known^15^. In the 20-year follow-up of WOSCOPS, the association between statin therapy and non-cardiovascular events in diabetics was investigated. Statin therapy was associated with fewer hospital admissions due to non-cardiovascular complications of diabetes. This study also found a unique association between statin therapy and risk of cataract which had not been previously reported.

One major limitation of this 20-year follow-up study is that over the latter 10 years there are no available data on statin therapy or other lipid lowering medications which could have been used by either group. As guidelines were rapidly changing in this time period regarding statin use and dosing, potential imbalances between the groups in this regard could have a significant impact on outcomes^7^. Another limitation of any trial of this duration is that baseline variables and risks within the population could have become unbalanced. Such examples include new smokers, new medications with drug interactions, and new disease states.

**Figure 3.**
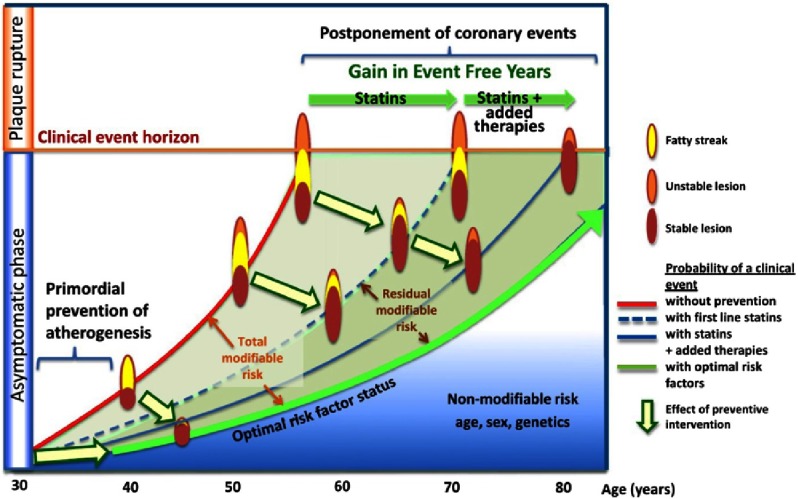
Trajectories in CHD prevention. Primary prevention of CHD in young adults with modifiable risk factors can change the trajectory of disease. Risk factor modification by statin therapy can lead to a significant gain in the event frees years and postponement of CHD events. Reproduced with permission from Dr. Packard and Colin McCowan.

Recently there has been more focus on modifying the lifetime risk from atherosclerotic disease. Young or middle-aged adults, who benefit from primary prevention therapy with statins, usually have low short-term risk. Therefore, researchers recommend focusing on the trajectory of disease and gains in event free years instead of short term event rates. Primary prevention with statins can potentially change the trajectory of atherosclerotic disease over a lifetime and result in a gain in event free years (Fig. 3)^16^.

As depicted in Fig. 3, despite the effect of statins in reducing the risk profile of individuals with modifiable risk factors for CHD, a significant residual risk persists despite the use of statin therapy. This may be attributable to the failure to achieve LDL-C levels low enough with statin therapy alone. Although statins are the first line agents for reducing LDL-C and therefore reducing risk of CHD, proprotein convertase subtilisin/kexin type 9 (PCSK9) inhibitors have been shown to significantly reduce the LDL-C^17^ in addition to statin therapy. Evolocumab is a PCSK9 inhibitor that was shown to significantly reduce LDL-C with or without high dose Atorvastatin^18^; similar potent LDL-C lowering from Evolocumab has been shown in individuals with muscle-related statin intolerance^19^. Results from ongoing outcome trials will provide more information on the safety and efficacy of PCSK9 inhibitors in low risk patients and in patients with known cardiovascular disease^20^. Treatment with PCSK9 inhibitors can potentially close the gap between LDL-C levels and ideal target thereby allowing patients to reach their optimal cardiovascular risk status. Ultimately, longer-term follow-up of these clinical trials will determine if the same legacy effect that applies to statins will apply to PCSK9 inhibitors.

At this stage PCSK9 inhibitors are very expensive and their long-term safety and efficacy remains unproven. However, one can envision a new paradigm of cardiovascular prevention for high risk patients in which a short-term of very aggressive LDL-C lowering with PCSK-9 inhibitors in addition to statin therapy is used to stabilize the atherosclerosis process at a vulnerable young age. This period could be followed by maintenance therapy with high intensity statins to stabilize existing plaque. Such a strategy may result in long-term prevention of cardiovascular events. Pending the long-term results of clinical trials, the proposed paradigm may provide a potent and cost-effective treatment for long-term cardiovascular risk reduction.^21^

## What have we learned?

The 20-year follow-up data from WOSCOPS provides clinical and safety evidence of long-term benefit of pravastatin therapy for primary prevention in individuals with hypercholesterolemia and no established CHD. The risk reduction in cardiovascular outcomes from 5 years of pravastatin therapy was persistent over the 20-year period and led to reduced mortality and hospitalizations. Pravastatin therapy was safe, cost-effective and was not associated with increased risk of non- cardiovascular events or cancer. This study provides evidence of a legacy benefit of primary prevention with pravastatin and adds to the current evidence available in favor of statin use in individuals without established CHD.
